# A Fatal Case of Immune Checkpoint Inhibitor‐Mediated Myasthenia Gravis, Myositis, and Cardiomyopathy Overlap Syndrome in Urothelial Carcinoma

**DOI:** 10.1002/cnr2.2140

**Published:** 2024-07-23

**Authors:** Matthew T. Newman, Mihir Bikhchandani

**Affiliations:** ^1^ Department of Internal Medicine Kaiser Permanente Los Angeles Medical Center Los Angeles California USA; ^2^ Department of Hematology & Oncology Kaiser Permanente Los Angeles Medical Center Los Angeles California USA

**Keywords:** cardiomyopathy, immune checkpoint inhibitors, immune‐related adverse events, myasthenia gravis, myositis

## Abstract

**Background:**

Immune checkpoint inhibitors (ICIs) have led to improved outcomes for many cancer types. However, their use can also precipitate immune‐related adverse events (irAEs) that can affect any organ system. While irAEs are often mild, they rarely affect multiple organ systems concurrently and can be fatal.

**Case:**

We report a fatal case of myasthenia gravis, myositis, and cardiotoxicity overlap syndrome precipitated by the ICI pembrolizumab along with a brief review of available literature.

**Conclusion:**

Early recognition of high grade irAEs and prompt intervention is essential. Despite the poor prognosis of these overlap syndromes, current recommendations offer little guidance for severe cases and warrant a call for increased awareness and expansion of available therapeutics.

**Abbreviations**
AChR: acetylcholine receptor antibodies
CK: creatine kinase
CT: computed tomography
CTLA‐4: cytotoxic T lymphocyte anitgen‐4
Cycle 1: C1
Cycle 2: C2
DNI: do not intubate
DNR: do not resuscitate
*HER2*
: human epidermal growth factor receptor 2
hsTrop: high sensitivity troponin
ICI: immune checkpoint inhibitors
ICI‐MG: immune checkpoint inhibitor‐related Myasthenia Gravis
irAE: immune‐related adverse events
IVIG: intravenous immunoglobulin
MGFA: myasthenia gravis foundation of America
MR: magnetic resonance
nAE: neurologic adverse events
PD‐1: programmed cell death‐1 antigen
PD‐L1: programmed cell ligand‐1
PET: positron emission tomography
pg/mL: picograms/milliliter
PLEX: plasma exchange
TTE: transthoracic echocardiogram
U/L: units/liter

## 1 Introduction

The discovery and application of immune checkpoint inhibitors (ICIs) has led to a paradigm shift in cancer care with unprecedented outcomes across many cancer types. Pembrolizumab, an ICI targeting programmed cell death‐1 (PD‐1) antigen, enables identification and destruction of neoplastic cells by the immune system by preventing interaction of this receptor with its ligand, programmed death ligand‐1 (PD‐L1)—a target that is often upregulated by tumor to evade detection. A powerful tool to reengage the immune system, pembrolizumab has been approved for use in many cancers, including advanced urothelial carcinoma. However, immune‐related adverse events (irAEs) representing off‐target activation of immune cells following ICI therapy are not uncommon and affect 40%–45% of patients on treatment with pembrolizumab [1]. irAEs include a broad spectrum of pathology and involve cutaneous, gastrointestinal, pulmonary, and hepatic systems most frequently [1].

Among those affected, a small number will experience neurologic adverse events (nAEs) following pembrolizumab, which complicate approximately 6% of cases in pooled analyses, with high grade nAEs comprising 0.2% [2]. Manifestations of high grade nAEs such as ICI‐related myasthenia gravis (ICI‐MG) can include serious complications such as respiratory compromise and dysphagia [2]. Fatal complications are more frequently reported in those presenting with concomitant ICI‐MG, myositis, and cardiomyopathy [1, 3]. Despite this, limited reports are available describing this overlap syndrome and effective therapies, which represents a significant knowledge gap in the setting of expanded approvals for pembrolizumab and other ICIs. Herein, we report a fatal case of ICI‐MG, myositis, and cardiomyopathy in a patient with advanced urothelial carcinoma treated with pembrolizumab.

## 2 Case Presentation

An 87‐year‐old male without autoimmune history was diagnosed with advanced urothelial carcinoma in May of 2023 following presentation for back pain and acute kidney injury. Computed tomography (CT) scan of the chest, abdomen, and pelvis revealed a left sided renal pelvis mass measuring 5.5 × 4.5 × 2.5 cm enveloping the left renal vein. A CT‐guided biopsy of the mass yielded invasive high‐grade urothelial carcinoma (*HER2* mutated). Subsequent positron emission tomography (PET) scan did not identify additional sites of concern for metastatic spread. He was evaluated by urology consultants who felt he had unresectable disease. Based on functional status and kidney dysfunction, he was ineligible for platinum‐based chemotherapy and began treatment with pembrolizumab monotherapy.

Cycle 1 (C1) of pembrolizumab was well tolerated. Immediately following cycle 2 (C2) of pembrolizumab, the patient reported diffuse, intolerable pain, and fatigue all over his body, unrelieved by over‐the‐counter analgesics, which prevented him from ambulating. The following day, he developed left sided ptosis that progressed to bilateral ptosis. The patient then presented to our hospital, Kaiser Permanente's Los Angeles Medical Center, where physical exam confirmed bilateral ptosis with minimal eye‐opening capability (about 1 mm bilaterally), alongside bilateral ophthalmoplegia affecting leftward traction. Further neurologic examination revealed an otherwise intact cranial nerve assessment. Strength examination was limited by pain but was preserved in his extremities alongside retained reflexes. Laboratory examination was notable for elevated creatine kinase (CK) to 6042 U/L as well as a high sensitivity troponin (hsTrop) elevation to 7549 pg/mL in the absence of cardiopulmonary symptoms. Electrocardiogram showed left axis deviation and minimal voltage criteria for left ventricular hypertrophy in the absence of ST‐segment changes concerning for ischemia.

**FIGURE 1:**
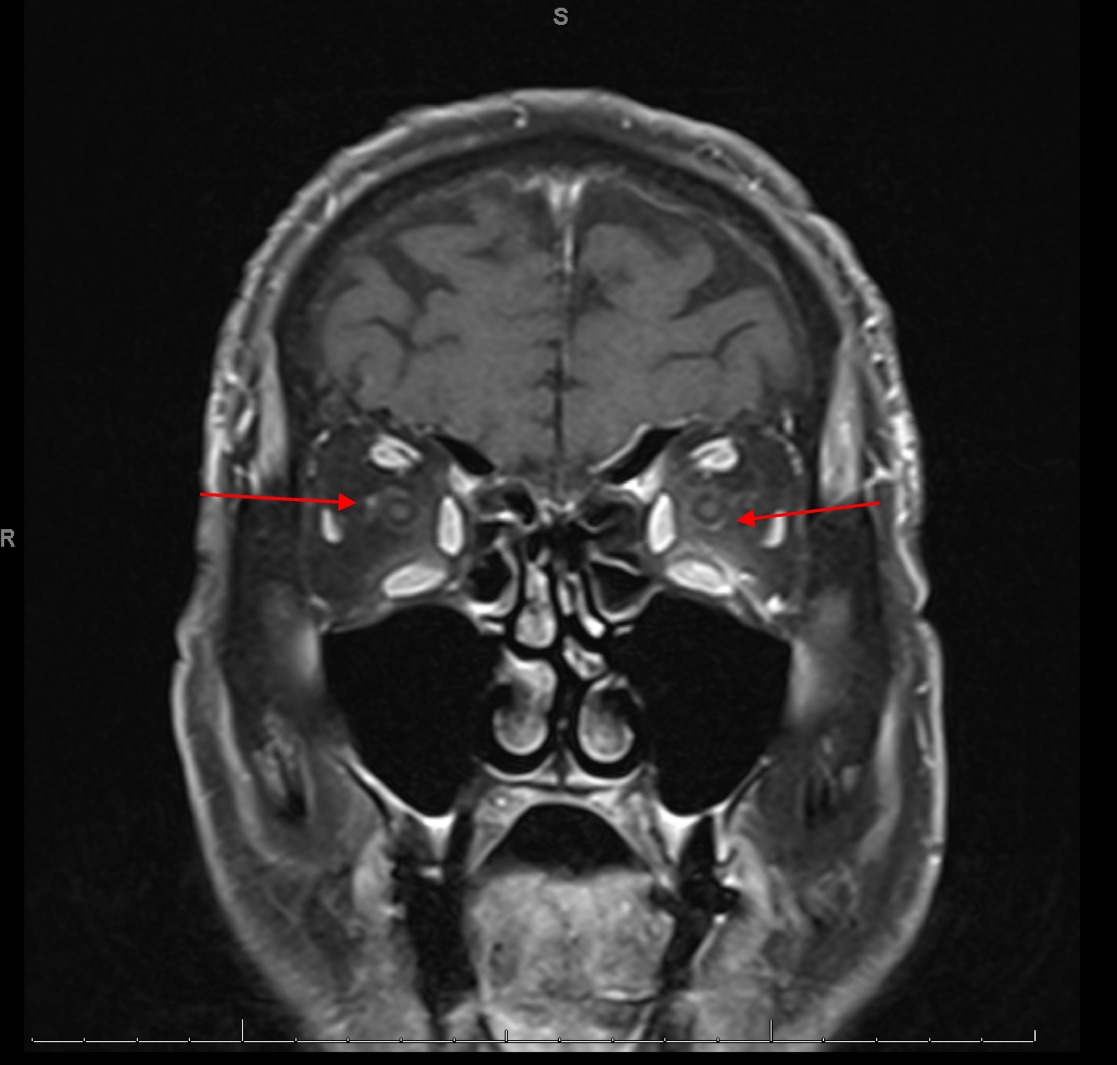
MR of the orbits demonstrating peri‐optic contrast enhancement of bilateral optic nerves.

His presentation was most compatible with an irAE secondary to pembrolizumab leading to diffuse myositis, cardiomyopathy, and ICI‐MG (ptosis, ophthalmoplegia). Further workup and treatment planning was coordinated with inpatient cardiology and neurology consultants. A transthoracic echocardiogram (TTE) showed mild concentric hypertrophy and hyperdynamic systolic function with an ejection fraction of 70%. An autoimmune antibody panel was drawn and was positive for striated muscle antibodies, a titer frequently seen in severe cases of myasthenia gravis, and negative for acetylcholine receptor (AChR) antibodies [4]. A magnetic resonance (MR) study of the orbits and brain was notable for peri‐optic enhancement of the bilateral optic nerve sheaths without leptomeningeal or cavernous sinus enhancement, which correlated with his bilateral traction deficit (Figure 1).

**FIGURE 2:**
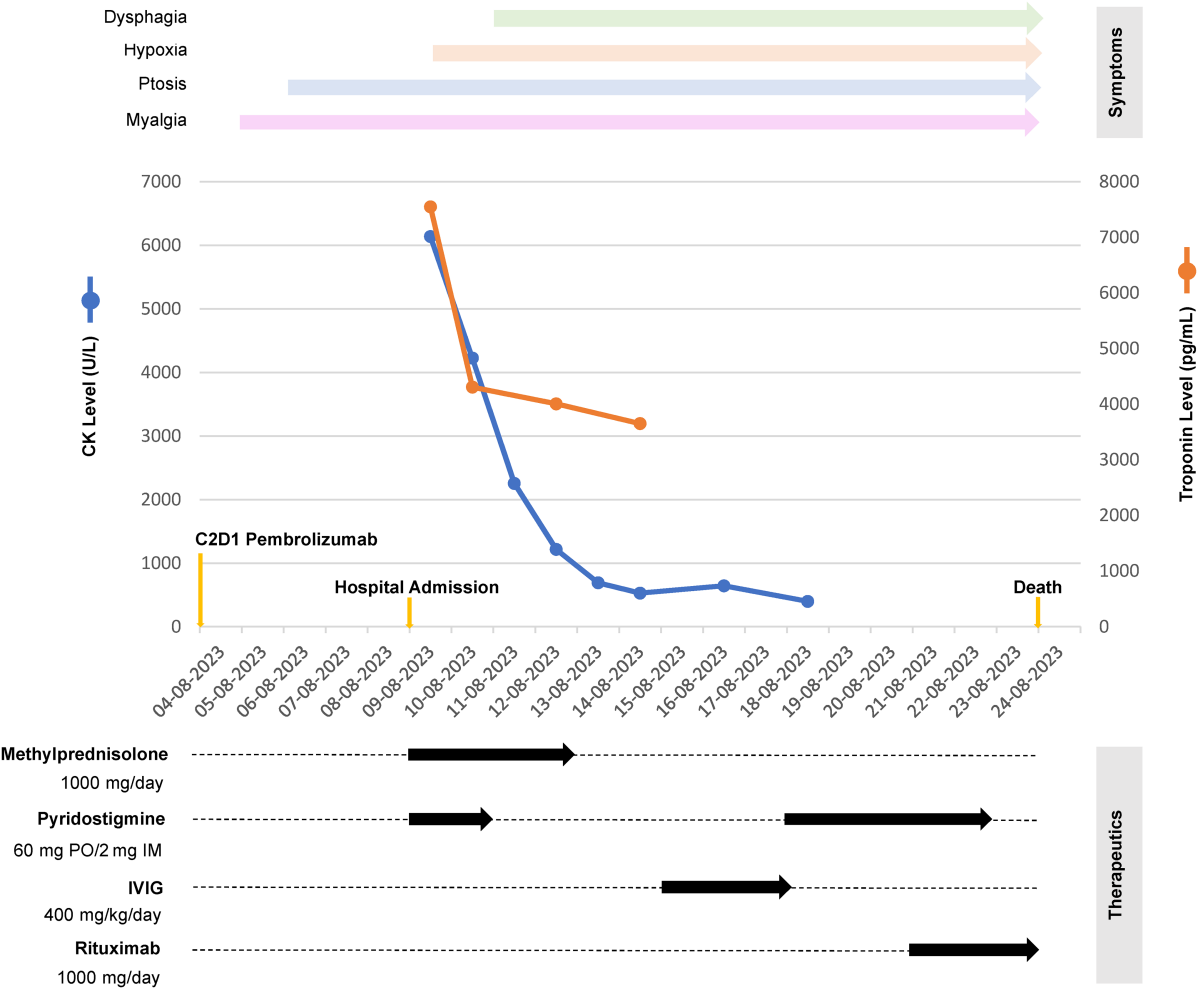
Time course of attempted therapeutic interventions and tracking of relevant lab values alongside progression of symptoms.

His initial course of therapeutics included pulse dose steroids (1 g of methylprednisolone/day), as well as pyridostigmine (60 mg, three times a day) on Day 1 of admission. The patient had gradual improvement in his myalgias alongside a downtrend in both his CK and hsTrop levels while on these therapies (Figure 2). However, no improvement was seen in the ptosis, and the ophthalmoplegia progressed to include traction deficits in all directions. On Day 3, he developed dysphagia and lost the ability to safely chew food and take oral medication. This persisted beyond the predetermined course of pyridostigmine and steroids, which were discontinued on Day 3 and 5, respectively, due to lack of improvement, as well as initial concern for increased aspiration risk related to muscarinic side effects.

A goals of care discussion was held with the patient and his family to determine the next phase of treatment. The patient decided that placement of a central line was not in line with his treatment goals, and the decision was made to start intravenous immunoglobulin (IVIG) therapy in lieu of plasma exchange (PLEX). He received 4 days of IVIG with no improvement in the ptosis, ophthalmoplegia, or dysphagia. He was then treated with a second course of pyridostigmine and finally rituximab. This was continued for four additional days without any improvement in his symptoms. On the morning of Day 16, his oxygen requirements increased, and he experienced progressive hypotension. As the patient was DNR/DNI without selective treatment, no further escalation of care was undertaken. He expired shortly thereafter on Day 16 with the cause of death believed to be aspiration.

## 3 Discussion

In summary, our case highlights severe complications affecting multiple organ systems concurrently that can result from checkpoint inhibitor therapy. By bringing attention to his clinical course and medical decision making, we hope to add to the continued discussion regarding effective management of this rare condition. We first focus on a review of ICI‐MG, as we believe our patient's precipitous decline and death was mainly attributed to this. We then expand our discussion to describe the overlap syndrome comprising of ICI‐MG, myositis, and myocarditis that our patient ultimately suffered from.

The most complete review of the literature has captured only 63 cases of ICI‐MG in patients across all ICI therapy [3]. This entity is unique in its severity compared to idiopathic MG, with the former leading to higher‐grade symptomatology compared the latter as measured by the Myasthenia Gravis Foundation of America (MGFA) scale [3]. Reported presentations among this complete cohort of patients included ptosis, dyspnea, and limb weakness most commonly, with dysphagia affecting less than half of patients [3]. Optic neuritis prevalence was not captured in this review, but another group has summarized 14 reported cases thus far [5]. Multisystem manifestations including myositis and cardiomyopathy are decidedly uncommon and have poorer prognoses, occurring in 8%–14% of reported ICI‐MG cases [1, 3].

The increasing prevalence of fatal ICI‐associated conditions has only been reported by a single study that captured fatal cases of myocarditis, some of which include concomitant ICI‐MG and myositis as seen in our case [6]. A smaller single center study published in 2022 sought to identify predictors of mortality in patients with this variant overlap syndrome. Troponin elevation, as seen in our patient, was the best predictor [7].

Despite recognition of this variant presentation, there are limited available therapeutics for these serious complications with current guideline recommendations for refractory cases beyond corticosteroids and pyridostigmine limited to rituximab and PLEX [8]. Sparse but promising data exists highlighting the prompt initiation of both IVIG and IV steroids to improve outcomes for overlap ICI‐MG, myositis, and cardiomyopathy [9]. Given our therapeutic choice of steroids and pyridostigmine several days prior to IVIG, treatment failure in our case supports further discussion of this aforementioned upfront immunosuppressive strategy beyond the case series level.

Though limited cases are available, some have also detailed successful use of abatacept (a cytotoxic T‐lymphocyte‐associated antigen 4 [CTLA‐4] agonist) to overcome severely refractory cases [10, 11]. This agent and other checkpoint agonists work in an obverse fashion to promote T cell energy through checkpoint activation, as opposed to inhibition as in ICIs. As current guidelines for refractory cases largely include nonspecific anti‐inflammatories (high dose steroids, IVIG) and antibody reducing agents (PLEX, rituximab), reversal of the strong and highly targeted effects of ICIs with a counteractive checkpoint agonist is an attractive approach in theory. To date, widespread implementation of targeted immune reversal agents such as abatacept or similar therapeutics for severe irAEs has not been pursued. Further investigation is warranted for checkpoint agonists in the treatment of refractory ICI‐MG overlap syndromes attributed to ICI‐therapy, which may pave the way for introduction of additional agents to target this severe and often fatal condition.

Taken together, we hope to bring attention and context to this rare and potentially fatal off‐target triad of ICI‐MG, myositis, and cardiotoxicity resulting from commonly used ICIs such as pembrolizumab, the incidence of which will likely increase in coming years in the setting of expanded approvals for similar agents. We believe these results to be highly clinically relevant for oncologists and their hospital medicine counterparts, who often work in conjunction to care for hospitalized patients with cancer, to help familiarize themselves with available therapeutics and to help navigate crucial interdisciplinary networks with in‐house consultants for management of this condition. Significant findings in this case included a rapid onset myasthenic and myositis prodrome following repeat ICI exposure, alongside precipitous clinical deterioration refractory to multiple lines of treatment without meaningful symptomatic improvement despite down‐trending of commonly utilized biochemical markers. Although the presence of more specific striated muscle autoantibodies in severe cases of MG has been recognized, high quality correlative studies tracking antibody titers with disease severity have not been performed, and as such lack of more specific biomarkers to assess treatment response in lieu of AChR antibodies presented a challenge [12]. Finally, as the patient's goals of care were not congruent with escalation to PLEX, we also cannot comment on this therapeutic's efficacy in this case. Despite this, treatment options for severe cases still remain limited to nonspecific agents that in this case were near exhausted without benefit for our patient. Additional reporting and research are needed to better address this gap in therapeutics, the progress for which has only been made at the case report level in the absence of evidence‐based clinical studies.

## Author Contributions

**Matthew T. Newman:** writing–original draft, conceptualization, investigation, writing–review and editing. **Mihir Bikhchandani:** conceptualization, investigation, writing–original draft, writing–review and editing, supervision.

## Ethics Statement

This case report study was approved by the Southern California Kaiser Permanente Institutional Compliance Office for publication.

## Consent

Consent for drafting and publication of this case report was obtained from the patient's next of kin.

## Conflicts of Interest

The authors declare no conflicts of interest.

## Data Availability

The data that support the findings of this study are available from the corresponding author upon reasonable request.
